# Creep Damage Assessment of Ex-Service 12% Cr Power Plant Steel Using Digital Image Correlation and Quantitative Microstructural Evaluation

**DOI:** 10.3390/ma12193106

**Published:** 2019-09-24

**Authors:** Melody van Rooyen, Thorsten Becker, Johan Westraadt, Genevéve Marx

**Affiliations:** 1 Department of Mechanical and Mechatronic Engineering, Stellenbosch University, Stellenbosch 7600, South Africa; 2 Centre for High Resolution Transmission Electron Microscopy, Nelson Mandela University, Summerstrand, Port Elizabeth 6001, South Africa

**Keywords:** creep damage, 12% Cr steel, digital image correlation (DIC), M_23_C_6_, laves phase, Zener-Hollomon

## Abstract

The lifetime of steam pipelines in long-term operation in coal-fired power plants are limited due to material damage that resulted from creep exposure. In the present study, the authors comparatively assess the damage of ex-service 12% Cr piping steel with varying degrees of exposure while using accelerated creep tests that employ digital image correlation (DIC) as well as microstructural investigation that is based on electron microscopy. The DIC technique, which allows multiple creep curves to be measured at temperatures ranging from 550–600 °C from a single specimen, revealed higher Zener–Hollomon parameters for a high damage material with a high void density when compared to a material with lower damage and lower void density. Both of the material states showed similar hardness values, subgrain sizes, and boundary character, despite the difference in void densities. Slightly higher inter-particle spacing of MX precipitates results in a lower threshold stress of 79 MPa for the high damage steel when compared to 97 MPa for the low damage material. Besides large Laves phase particles (>0.2 µm) that are found in the higher damaged materials that result in solid solution depletion, the most prominent microstructural damage indicator was a lower density of M_23_C_6_ precipitates. Therefore, the observations indicate that the Zener–Hollomon parameter and M_23_C_6_ particles are good damage assessment indicators between the most extreme damage states and they predict a lower damage level for a medium void density material.

## 1. Introduction

The reliable operation of thermal power stations is largely tied to the material integrity and life management of critical components. The material properties are degraded through several processes, including creep, as these components are subjected to high operating temperatures and stresses over extended service times (>20 years). Within the power industry, the tempered martensitic ferritic 12% Cr steel, known as X20CrMoV12-1 (X20 hereafter) under DIN designation, is well-known. Being considered a vintage alloy in the 9%–12% Cr steel group, X20 has been in service as main steam piping in coal-fired power plants for over 60 years [1]. However, there is continued interest in this steel, as it is currently in use in about half of the power stations in South Africa and, in some cases, has outperformed its replacement P91 (X10CrMoVNb9-1) in terms of damage accumulation [2].

Several methodologies are available for characterising creep damage [3,4]. These methods commonly focus on two closely related aspects, namely, (i) the creep deformation or strength behavior (revealed through mechanical testing) and (ii) microstructural and damage evolution (revealed through metallographic methods).

There have been various approaches to characterising the extent of material degradation in X20 and other 9%–12% Cr steels through mechanical testing. Masuyama [5] proposed a creep life fraction model that is based on the reduction of hardness displayed by interrupted creep samples of 9% Cr steel. Such hardness drop is, in turn, linked to microstructural components, such as the reduction in dislocation density resulting from stress-driven subgrain growth, an increase in creep cavity density [6] and coarsening of carbides [7]. Although this method allows for practical degradation assessment during service of power plant steels, hardness measurements are sensitive to material processing parameters and heterogeneous microstructural features [5,8].

High temperature tensile tests have revealed a decreased strength and ductility of exposed X20, although the property differences can be concealed by different initial heat treatments [8,9]. El Rayes and El-Danaf [10] found that constant strain rate tensile tests at elevated temperatures revealed minor differences between the deformation mechanisms of retired and as-produced 12% Cr steel through the calculation of stress exponents and activation energies after correcting for the influence of threshold stresses. These stresses arise from the attractive force between the MX carbonitride particles and detaching dislocations and they are themselves dependent on material degradation through the coarsening of precipitates [11,12]. However, typically, the creep rate-controlling mechanisms of 9%–12% Cr steels are often investigated by means of conventional creep testing rigs, which involve long testing times (>10,000 h) due to the slow creep rates that are associated with diffusional creep mechanisms experienced at service conditions.

Traditional tests use single samples at a single uniform temperature and they can be costly due to the heating requirements of traditional furnaces as well as the continuous monitoring that is required for strain measurement. Furthermore, an average strain measurement is obtained using conventional equipment, such as strain gages and LVDTs. As a result, only one specimen can be used to simulate one stress and one temperature condition at a time.

With the increasing popularity of Digital Image Correlation (DIC) as a strain measurement tool for material testing, deformation data can be collected in a full-field manner [13]. This non-contact technique correlates the deformation of a speckle pattern on a material surface during loading while using digital image recording, resulting in a deformation map from which a strain map can be derived. Spatial resolution for DIC is determined by the size of interrogation windows (or subsets) that make up the Region of Interest (ROI) and they are usually in the order of 10–100 pixels [14]. Sakanashi et al. [15] extended the use of DIC to measure creep deformation spatially across a stainless steel weldment. In the authors’ previous work, DIC was successfully used to measure tensile properties of X20 at several elevated temperatures from a single sample that was subjected to a thermal gradient while using resistive heating in a thermomechanical simulator [16].

It is clear that the mechanical properties of ex-service 9%–12% Cr steels is closely related to their microstructural stability during long-term creep exposure. The most commonly practiced damage surveying method is replica metallography, which monitors creep cavity density that can be directly linked to remnant material life [17]. However, this technique suffers from several limitations [4]: it is only restricted to the surface of the pipe; void formation from the replica preparation results in spurious measurements; an incorrect judgment of damage can result from an apparent reduction in void density due to coalescence nearing the end of component life; and, other forms of microstructural degradation can mask the effects of cavity growth [6]. In some cases [2], X20 piping has not manifested damage in terms of creep cavitation up to 200,000 hours of service exposure, which requires an alternative means of microstructural damage assessment. 

Therefore, microstructural investigations are necessary, and focus is usually placed on three main microstructural barriers to creep deformation, namely, micro-grains (including subgrains), second-phase precipitates, and dislocation networks. Long term (>130,000 h) creep tests on X20 have revealed a coarsening of M_23_C_6_ carbides, precipitation of Mo-rich Laves phase, a reduction in dislocation densities and concomitant coarsening of subgrains while using Scanning Electron Microscopy (SEM), Transmission SEM (TEM), and Electron Backscatter Diffraction (EBSD) [18,19]. However, there have been few attempts to compare the changes of the microstructural details in ex-service material in a quantitative manner. Hu et al. [7] proposed the change in the Cr:Fe ratio in carbides with operating time to be an index of life depletion for X20 superheater tubes. Straub et al. [8] found little difference between the dislocation density, subgrain size, and cavity density of creep exposed tube as compared to an initial melt, although a coarsening of precipitates was identified, quantitatively demonstrated later by Hu et al. [9] while using TEM on extraction replicas. Other investigations [20] of ex-service X20 displayed a lack of creep cavitation, but a reduction in subgrain-interior dislocation densities. This suggests that certain microstructural features are more suitable indicators of creep damage than others. Indeed, some microstructural or creep cavity distribution factors are more sensitive indicators of damage as the rate of damage development varies, depending on the extent of life exhaustion [21].

It is not clear what microstructural-based technique is best suited to the damage assessment of ex-service material due to the complexity of creep in 12% Cr steels. A consensus can be reached on the most prominent damage indicator by using accelerated creep tests (<10 h testing time) and exploring the exposure-driven microstructural features that explain the resulting deformation behavior in a comparative manner between X20 with varying levels of pre-existing damage. This work in no way attempts to use accelerated testing (at higher stresses) to extrapolate to the service conditions of the piping material (at lower stresses) due to the difficulty in accounting for the stress state sensitivities of damage micro-mechanisms [22]. Instead, the deformation behavior at accelerated conditions is used as a means of assessing the loss of creep resistance between the material states. By using the developed DIC technique [23], material requirements are reduced, as multiple deformation curves at several temperatures can be conveniently measured from a single sample. Therefore, the present work aims to assess the creep damage of various ex-service X20 piping material in a comparative manner while using a combination of post-exposure mechanical deformation tests and microstructural evaluation.

A Gleeble thermo-mechanical simulator allows for the generation of a near-parabolic shaped temperature profile longitudinally across the specimen through resistive heating. Multiple accelerated creep curves are, in this way, generated from a single specimen and they are captured by DIC. Following testing, previously explored [23,24] electron microscopy techniques were employed to holistically classify the damage levels of the tested samples across various microstructural indicators, including: boundary character from EBSD; substructure (dislocations and micro-grains) and large precipitate details using Concentric Backscatter (CBS) SEM, dislocation density details from an Annular Dark Field Scanning TEM (ADF-STEM); and, smaller precipitate morphology identified by Energy-Filtered TEM (EFTEM). 

In what follows, a brief description of the materials, selection of sample geometry, and experimental procedure for the DIC testing and microstructural study is supplied. This is followed by a presentation of the accelerated creep behaviour, hardness measurements, and comparison of microstructural elements. Finally, a discussion is provided linking the two types of damage assessment, offering a conclusion on the plausibility of the DIC technique and suggested microstructural feature as damage indicators. 

## 2. Experimental Details

### 2.1. Materials

Table 1 provides the chemical composition of the virgin X20, as measured by inductively coupled plasma, optical emission spectroscopy, and combustion analysis. 

Table 2 gives the operating conditions of the ex-service material. EXL, EXM, and EXH stand for “Low”, “Medium”, and “High” damaged ex-service X20 material, respectively, as categorised by replica-based cavity density measurements.

### 2.2. Specimen Geometry

**Figure 1:**
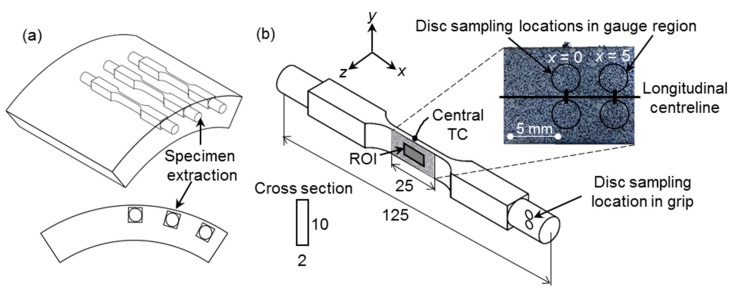
Illustration of the (**a**) sampling scheme in piping material and the (**b**) selected geometry for accelerated creep tests as well as the location of the disc specimens extracted for microstructural analysis of the gauge and grip regions. All dimensions are in mm. ROI = Region of Interest. TC = thermocouple. *x* = 0 indicates the specimen center.

The test specimens were machined from virgin and ex-service X20 main steam piping sections provided by a local power utility with internal diameters of 255 ± 5 mm and wall thicknesses of 35 ± 1 mm. All of the specimens were axially oriented with respect to the pipe orientation, as shown in Figure 1a and they are located close to the pipe outer diameter.

Previous investigations [16,23] have shown that the Gleeble specimen geometry has a direct influence on the resulting temperature distribution and, consequently, the stress distribution. In this work, the selected specimen geometry has a 2 × 10 mm^2^ (shown in Figure 1b) cross-sectional area that differs from the traditional 10 × 10 mm^2^ Gleeble specimen, respectively, called flat and square specimens. Results from the square specimens were included for comparative purposes. Note the coordinate system in Figure 1b, where *x* is the longitudinal loading direction and *y* is the transverse direction. For both geometries, the *x*-direction temperature profiles can be represented by a second-order polynomial [25]. 

For the microstructural analysis, 3 mm diameter discs were wire cut from the specimens at three locations: from the grips; near the gauge center (corresponding to the maximum temperature of 600 °C) and at an offset of about 5 mm from the center (subjected to about 550 °C), as indicated in Figure 1b.

### 2.3. Accelerated Creep Test Setup

The specimens were tested in the vacuum chamber of a Gleeble 3800 thermomechanical loading system (Dynamic Systems Inc., New York, NY, USA) while using a technique that was previously presented by the authors [16,23]. Resistive heating through feedback control from a K-type thermocouple spot-welded to the specimen center (see Figure 1b) resulted in a temperature profile that quadratically decreases from a maximum in the specimen center towards the water-cooled grips along the specimen axis. The full-field temperature map was measured by a Fluke Ti400 (Fluke Corporation, Everett, WA, USA) infrared (IR) camera on the rear specimen surface, which was coated with a highly emissive and heat resistant black paint. The quadratic temperature profile varied from 600 °C in the center (*x* = 0) to about 550 °C symmetrically over 5 mm. The grips were subjected to room temperatures and minimal strain due to the inherent Gleeble setup, and are thereby considered to represent the bulk material. 

**Figure 2:**
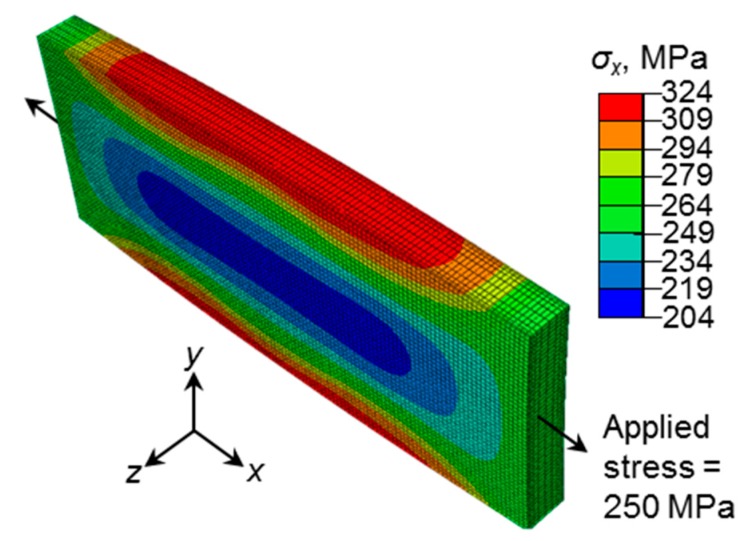
Distribution of *x* direction stresses (*σ_x_*) from a finite element (FE) analysis of the flat specimen gauge region at an applied stress of 250 MPa.

The thermal profile will influence the distribution of stress in the specimen gauge region as a direct result of non-uniform thermal expansion. To investigate this effect, a finite element (FE) elastic-plastic model was created for the gauge region while using ABAQUS (version 2017, Dassault Systèmes Simulia Corp., Johnston, RI, USA) FE software. Material parameters and model details used in the FE analysis can be found in [16] and [23]. Figure 2 reveals the distribution of stresses in the loading direction at an applied (engineering) stress of 250 MPa, showing a non-uniform stress region that results from the variable expansion effect of a non-uniform thermal profile. This expansion results in compressive stresses that reduce the applied stresses to lower values, particularly near the specimen gauge center, as noted in Figure 2. A narrow region that was centred about the gauge region develops, within which the stress remains uniform. Although not shown here, the square specimens resulted in a more gradual thermal profile (600–580 °C over 5 mm), which involves lower compressive stresses [23]. As a result, larger tensile stresses develop within these specimens when compared to its flat counterpart that is subjected to the same applied stress. 

By calculating the longitudinal variation of stress triaxiality (ratio of hydrostatic stress to von Mises stress) [26] over this region, as summarised in Table 3, the stress distribution can be analysed for its alignment with the ideal uniaxial stress condition. A ROI of 10 × 5 (*x* × *y*) mm^2^ centred on the gauge area was selected for DIC processing, as the triaxiality values within this area are near 0.33, which is typical of uniaxial stress states. Through restriction of the ROI to the uniform stress region within the specimen gauge, the issue of strain measurement from a non-uniform stress field superimposed onto the parabolic temperature profile is avoided. The actual stress range in the ROI is also reported in Table 3. Hereafter, the average actual stress *σ_x_* over the ROI is reported. 

Displacement measurement maps were obtained by a two-camera LaVision DIC systemthat consists of 5 megapixel 12 bit cameras paired with 75 mm macro lenses that resulted in a 60 mm × 50 mm field of view and 40 pixel/mm image scaling. After the temperature field had stabilised, the specimens were subjected to a constant load, whilst images of a heat-resistant speckle pattern painted over the 25 mm × 10 mm gauge area (as shown in Figure 1b) were recorded through the vacuum chamber viewing window at a rate of 0.1 Hz. Blue colour filters and high-powered white LEDs were used to suppress surface radiation noise. 

Analysis of images captured during testing was performed while using the LaVision DaVis 8.4 correlation software over the ROI with a 61 pixel^2^ subset size with a 15 pixel overlap that resulted in a software-estimated spatial resolution of 0.4 mm and a strain accuracy of 0.05%. Subsequently, a piece-wise quadratic polynomial surface fit was applied to the DIC displacement data while using MATLAB (version 2019a, MathWorks, Natick, MA, USA) across a window size of seven data points. The creep strain rate was obtained by applying a moving average filter to the creep strain-time data over a sliding window size of 30–40 points. Further details surrounding the DIC technique are provided in [23]. Thereore, creep strain measured at a position within the ROI of the specimen gauge section is associated with a specific temperature, as measured by the IR imager, and an average longitudinal stress with near-uniaxial triaxiality ratios, as calculated from FE analysis. 

Following testing, Vickers hardness measurements were made along the longitudinal direction of the flat specimen gauge regions and on the grips using a 1 kg load and 10 s holding time according to ASTM E834-11 [27].

### 2.4. Deformation Mechanism Regime

Accelerated creep testing in this work was conducted at actual stresses that ranged from 145 to 260 MPa and with temperatures that ranged from 550 to 600 °C. Given this range of testing stresses and temperatures, it may be worthwhile to elucidate the most likely dominant mechanism while using deformation mechanism maps. As literature pertaining to map construction for virgin X20 is scarce and almost non-existent for service-exposed materials, approximate maps were used here based on deformation modes that were encountered by a similar ferritic-martensitic steels, namely F82H [28], for the range of normalised temperatures and stresses that were used in the current work. Based on these mechanism maps, it is expected that accelerated creep tests will be within the low temperature dislocation climb regime with creep rates that vary from 10^−5^/s to 10^−3^/s. 

### 2.5. Microstructural Examination

Bulk specimen discs were sectioned from the tested samples (at locations indicated in Figure 1b) and polished to a 0.25 µm colloidal silica finish. Backscatter Electron SEM (BS-SEM) was employed to measure initial cavity densities of the untested pipe sections using ImageJ [29] image processing software (version 2.0.0-rc-59/1.52p, National Institutes of Health, Bethesda, MD, USA),whilst subgrain details of the gauge samples were qualitatively analysed. The EXL and EXH material was selected for more qualitative TEM, STEM, and Transmission Kikuchi Diffraction (TKD) study in order to isolate the largest differences in damage features. Subsequently, thin foils were prepared from the discs using a Struers TenuPol-5 (Struers A/S, Ballerup, Denmark) twin-jet electropolisher (5% HCIO_4_ solution; 21–30 V; −20 °C). 

The micro-grains, M_23_C_6_ carbides, and Laves phase precipitates were investigated while using a CBS detector that was fitted to a FEI Helios NanoLab DualBeam 650 Focused Ion Beam (FIB)-SEM (Thermo Fisher Scientific, Waltham, MA, USA) operated at a 5 kV accelerating voltage, 0.20 nA probe current and 4 mm working distance (WD). The identification of MX precipitates was facilitated while using Cr and V elemental maps that were acquired with the three-window method using EFTEM with a GATAN Quantum GIF fitted to a JEOL JEM 2100 LaB_6_ TEM (200 kV and 10 mRad collection angle). Analysis of micro-grain size was realised with the MIPAR [30] image analysis software (version 3.0.3, Ohio State University, Columbus, OH, USA) by measuring the short widths of the grains from the minor axes of fitted ellipses. The precipitates were identified based on the brightness variations in the CBS-SEM images, which are directly linked to the atomic number of constituent elements. Stereological corrections were applied to correct for smaller measured diameters due to sectioning of the M_23_C_6_ carbides and Laves-phase precipitates in the thin foil specimens [31,32]. 

Qualitative details regarding the distribution of dislocations in the X20 substructure was revealed through ADF-STEM on the same TEM used for the EFTEM analysis with inner and outer detector angles of 18 ± 5 mrad and 47 ± 5 mrad, respectively.

As it is challenging to distinguish between different grain boundaries while using imaging techniques alone, the boundary character of micro-grains is also studied through EBSD using a Nordlys HKL system of a JEOL JSM 7001F Field Emission Gun (FEG) SEM (JEOL Ltd., Tokyo, Japan) using settings of 15 keV, 4 nA, 22 mm WD, and 0.2 µm step size. This step size enables the identification of individual micro-grains (with widths varying from 0.7–1.2 µm [18]) in a statistically significant manner. In accordance with the definition that was provided by Peŝiĉka et al. [33], the term “micro-grains” is used hereafter and it refers to the finest features of tempered martensite ferritic steels that consist of subgrains (with misorientations ~ 1°) and small grains with high misorientation angles with adjacent small grains (here restricted to 2 –5° misorientations). This differentiation is needed due to the angular resolution limitations of orientation mapping, which can result in spurious subgrain measurements [19]. 

Post-processing on the EBSD data was performed while using the MTEX 5.2 MATLAB R2019a toolbox [34]. Prior to the processing of EBSD data, noise removal was achieved through a half quadratic optimization filter with a smoothing parameter of 0.2 and edge preservation was assured for boundaries above a 1.3° misorientation to retain relatively low angle boundaries. The grains were constructed with a 1.5° boundary misorientation and only grains larger than 5 pixels^2^ (about 0.64 µm^2^) were considered. No noise removal was performed for misorientation analysis to prevent smoothing over subgrain boundaries. 

Furthermore, EBSD allows for the reconstruction of prior-austenite grains into packets that are subdivided into blocks consisting of laths each typically formed from two different variants. A MTEX-based prior-austenite reconstruction graphical user interface while using a Markov clustering algorithm with an inflation power of 1.6 was used to identify block, packet, and prior-austenite grain boundaries (PAGB) [35].

To more accurately resolve micro-grains, TKD was combined with Energy Dispersive X-ray Spectroscopy (EDS) while using an Oxford X-Max EDS SDD detector. A step size of 0,03 µm allows for higher spatial resolution, whilst settings of 30 kV, 4 nA, 10 mm WD, and −20° specimen tilt were employed. 

## 3. Results

### 3.1. Accelerated Creep Deformation

**Figure 3:**
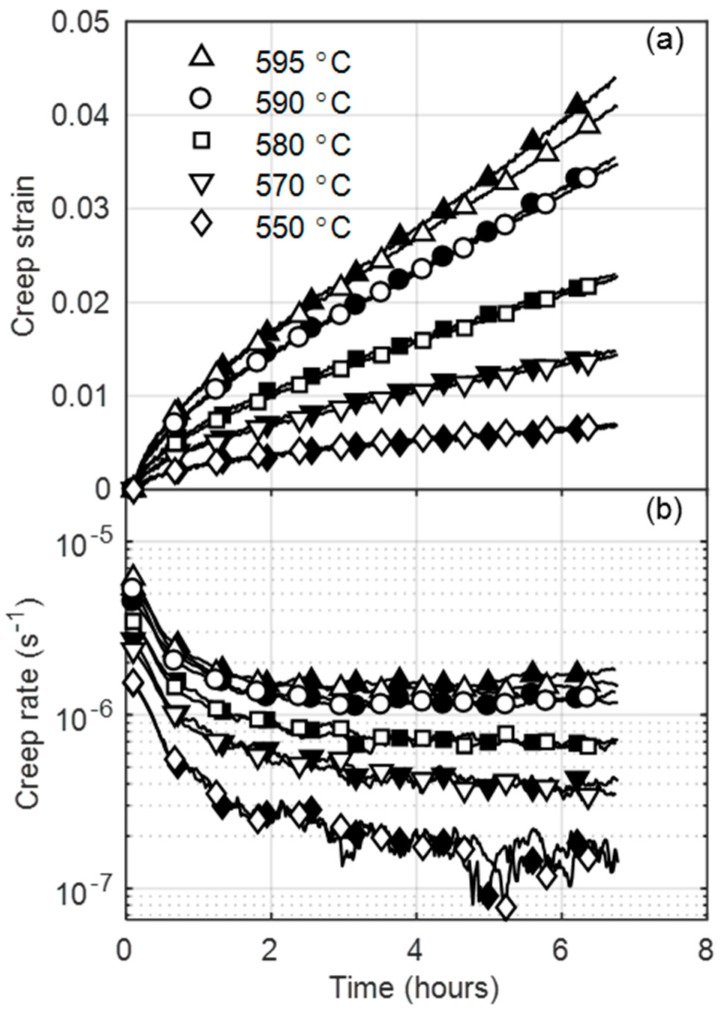
Evolution of accelerated (**a**) creep strain and (**b**) creep rate over time for two different (clear and shaded symbols) virgin X20 specimens at several temperatures.

Figure 3a shows creep strain-time curves at several temperatures at a 250 MPa applied stress for virgin X20. 

Good repeatability in measurements is indicated by minor differences between the two specimens, which is better demonstrated by the variation of the logarithm of creep rate with time plotted in Figure 3b. At higher temperatures (>580 °C), the curves consist of a primary creep stage with a decreasing creep rate, followed by a secondary stage with a minimum creep rate turning point, leading into accelerating rates that are caused by specimen necking in the tertiary stage. As the temperature decreases, the creep strains and rates decrease. For this reason, curves at temperatures below 580 °C remain within the primary stage for the virgin material. At lower temperatures (±550 °C), scatter in strain rate measurements is shown to increase with time. This is linked to the higher scatter that is assocaited with the temperature profile at higher distances from the control thermocouple located at the specimen center. Temeperature scatter, together with the higher relative noise associated with smaller strains, is amplified in the strain rate calculated due to the numerical differentiation (moving slope) scheme employed. 

**Figure 4:**
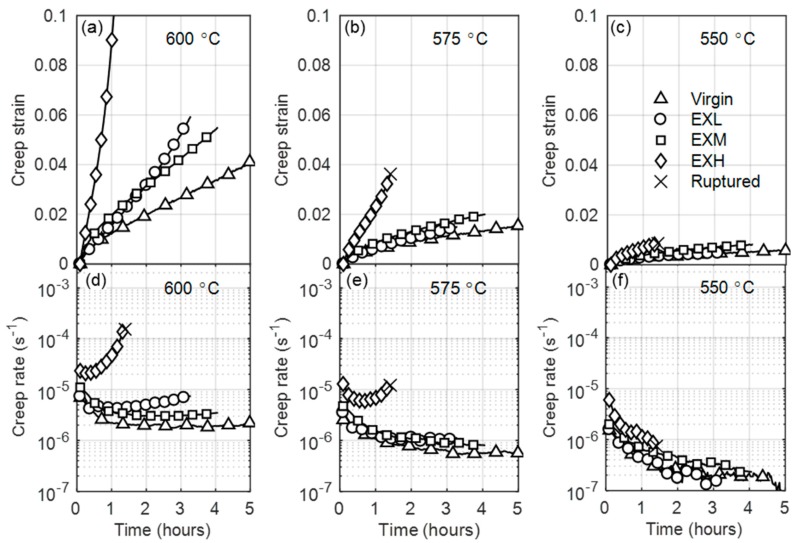
Comparison between the creep strains (**a**–**c**)) and creep rates (**d**–**f**)) of virgin and service-exposed material at 600 °C, 575 °C, and 550 °C for column 1, 2, and 3, respectively, for an applied stress of 250 MPa. Legend is given in (**c**).

Figure 4 shows a comparison between the creep behavior of various degrees of service-exposed and virgin material at different temperatures. Generally, higher strain rates are observed for the service-exposed material, resulting in more pronounced creep curve stages. This implies that the secondary creep stage is reached sooner with the same testing conditions as for virgin material. At 600 °C, the highly aged material displays a tertiary-dominated creep curve and it ruptures within 1 h of testing. At temperatures below 575 °C, creep strains remain within the primary stage for all material states, as evident from the lack of an turning point in Figure 4c for 550 °C. Highlighted are the accelerated creep rates when comparing EXL and EXM, which is discussed later.

**Figure 5:**
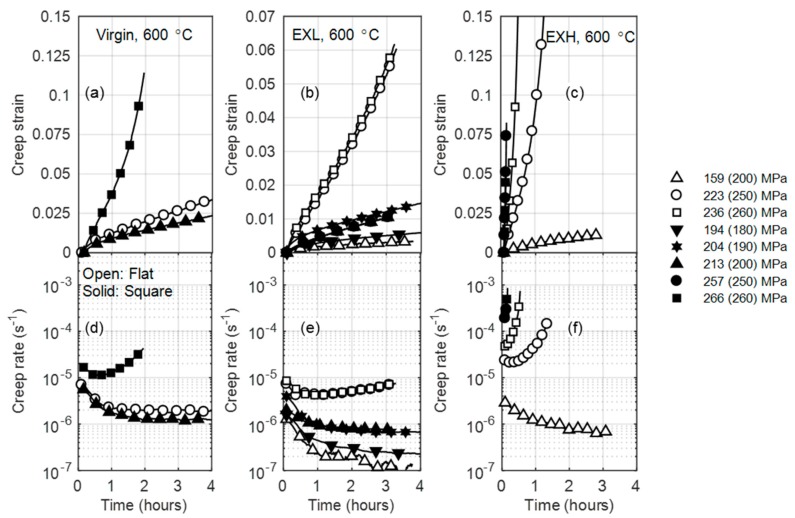
Creep strain and creep rate curves for virgin (**a**,**d**), EXL—Low damage ex-service (**b**,**e**), and EXH—High damage ex-service (**c**,**f**) material at 600 °C and various stresses. Open and solid symbols refer to the flat (2 × 10 mm^2^) and standard square (10 × 10 mm^2^) geometries, respectively. True stresses are reported (with applied stress in brackets) in the legend.

Figure 5 shows variations in creep strain and creep rate with stress of the virgin (a) and service-exposed material (b–d). For comparison, standard square Gleeble specimen data is included. Applied stress values are given in brackets next to actual, FE-determined stresses in the legend in Figure 5. Creep rates increase with stress, with EXL and EXH displaying increasingly higher strains and rates when compared to the material. Futhermore, higher creep strains and rates are observed for the square geometries than the flat specimens at the same applied stress. This is an outcome of the counteraction of the applied stress by lower compressive stresses (resulting from the more gradual temperature profile of the square specimens), leading to higher actual tensile stresses and correspondingly higher strain rates. 

### 3.2. Longitudinal Hardness Profiles

**Figure 6:**
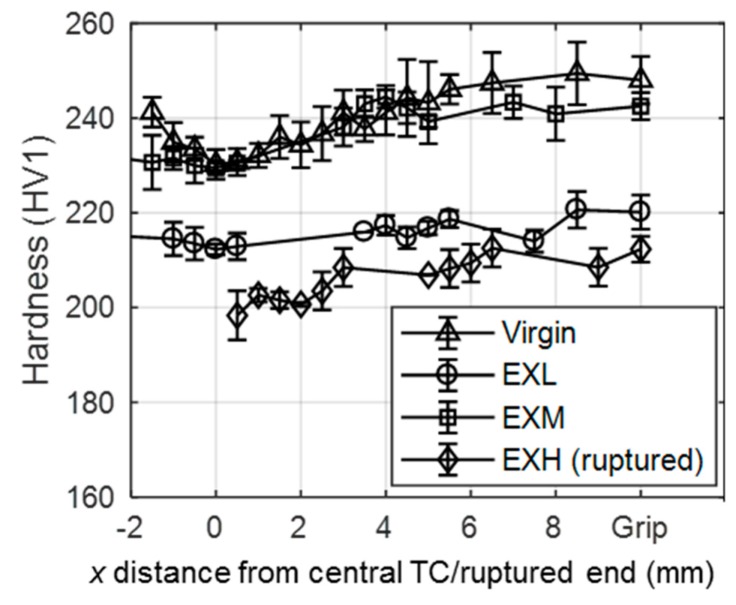
Variation of hardness with distance from the central thermocouple for the virgin, EXL, and EXM material, and with distance from the ruptured end of the EXH material.

Figure 6 shows that both service-exposed material and virgin material demonstrate a decrease in hardness towards centre of the specimens near the maximum temperature of 600 °C. When compared to the grip hardness readings, the drop in gauge hardness is attributed to the material age [5]. Similarly, a difference between EXL and EXM hardness readings is highlighted.

### 3.3. Microstructural Characterisation

The microstructural characterisation is predominantly based on the EXL and EXH states that theoretically should identify the largest difference in the microstructural features. Some reference is made to EXM, as previously highlighted. Its relevance is discussed later. 

#### 3.3.1. Comparison of Cavity Distributions

**Figure 7:**
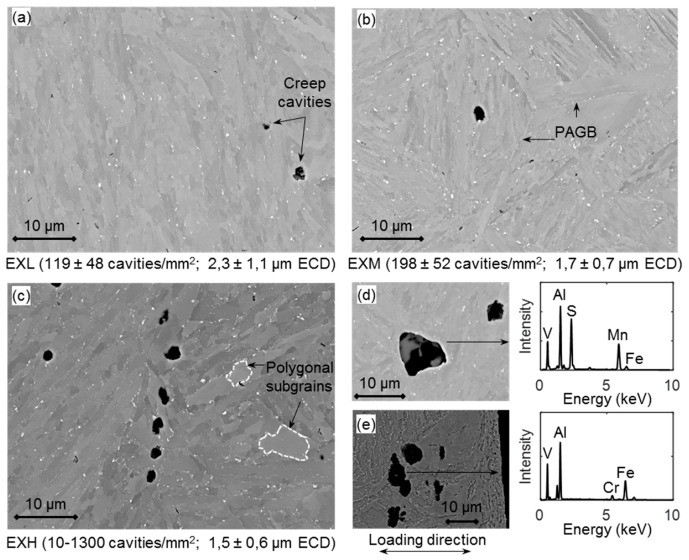
Backscatter Electron Scanning Electron Microscopy (BS-SEM) images showing micro-grains and voids in (**a**) EXL, (**b**) EXM and (**c**) EXH material. Large inclusions in (**d**) EXL material are shown by Energy Dispersive X-ray Spectroscopy (EDS) to contain Al and Mn and they are also located (**e**) near the ruptured end of the EXH material. ECD = Equivalent Circle Diameter.

BS-SEM images at 400× magnification taken on ex-service material pipe offcuts were subjected to a threshold adjustment in ImageJ, after which cavities with an equivalent circle diameter (ECD = $\sqrt{4\times area/\pi }$) larger than 0.1 µm, but less than 5 µm were counted. This allows for a direct comparison with surface replication results acquired while using optical microscopy with limited spatial resolution whilst excluding large pores from which inclusions have been removed during specimen preparation [8,36,37]. Qualitative differences in cavity distributions (shown as black speckles), large precipitates (white speckles), and micro-grains can be identified in 2000× magnification SEM micrographs in Figure 7, together with measured cavity details. 

Although not shown here, the cavity sizes are lognormally distributed. Interestingly, for the sampled material larger cavity sizes were measured for EXL than EXM and EXH—suggesting delayed cavity growth in the latter material possibly due to the production from a different melt having differences in virgin material void sizes and the presence of inclusions as nucleation sites [8]. Some large voids (>10 µm) in the EXL material are associated with inclusions, as suggested by EDS analysis in Figure 7d to be MnS and Al_2_O_3_ [8]. Larger void sizes could also partly explain the lower hardness measured for EXL as compared to EXM. However, in agreement with replica results, a wide range of cavity densities are recorded in the EXH material. Unlike in work reported by Aghajani et al. [18], the service-exposed material did not demonstrate significant cavitation on PAGBs in this orientation (one of which is indicated in Figure 7b). Instead, large intragranular cavities appear to be more common, mostly nucleating at large inclusion particles and coarse precipitates on micro-grain boundaries. An example of a cavity chain is shown for the EXH material in Figure 7c, which has the potential, with further creep exposure, to grow and merge to form micro-cracks, as has occurred due to inclusions after accelerated creep testing at 223 MPa in Figure 7e [37]. 

#### 3.3.2. Boundary Character from EBSD

**Figure 8:**
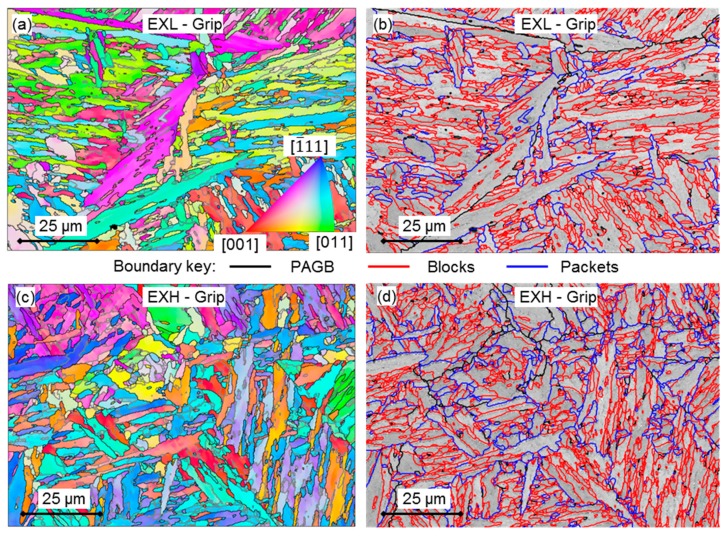
Inverse Pole Figures (IPF) maps of the tested (**a**) EXL and (**c**) EXH grip regions where the colouring identifies the crystallographic directions parallel to the sample normal (*z* direction) according to the colour key given in (**a**). Boundaries of >1.5° misorientaitons are outlined. These maps are used to reconstruct block (red), packet (blue), and PAGB (thick black) boundaries in (**b**,**d**), respectively.

Figure 8a,c show the Inverse Pole Figures (IPF) maps for the grip regions of the EXL and EXH specimens, respectively. No significant differences were observed between the grip and gauge regions. Prior-austenite reconstruction of the data allows for the determination of packet, block, and PAG boundaries, as shown in Figure 8b and d for EXL and EXH, respectively. 

Boundaries of micro-grains are difficult to distinguish from block boundaries due to the sampling size that was used for EBSD analysis (0.2 µm step size, aged micro-grain width < 0.8 µm [38]). However, as shown in the reconstructed maps, focus is placed on differences between block (red outlines), packets (blue), and continuous PAGB (thick black). As suggested by the work of Payton et al. [39], the extent of creep exposure can be identified by considering the densities of micro-grain, block and packet boundaries (length per unit area), which is summarised in Table 4 for the EXL and EXH material. 

From the above analysis, EXL demonstrates a decrease in micro-grain, block, and packet boundary density as compared to EXH, which suggested higher damage accumulation in the former material through migration of boundaries and grain growth. However, micro-grain boundary analysis using EBSD is subject to possible error, due to many virtual grains being detected from small misorientations between sub-block boundaries that are within the angular resolution of the EBSD technique [39]. A strong variant texture was also noted in the sampled PAG (possibly due to the pipe processing of the material [40]), where variants 1–6 occurred more frequently than other variants, resulting in the increased detection of block boundaries.

**Figure 9:**
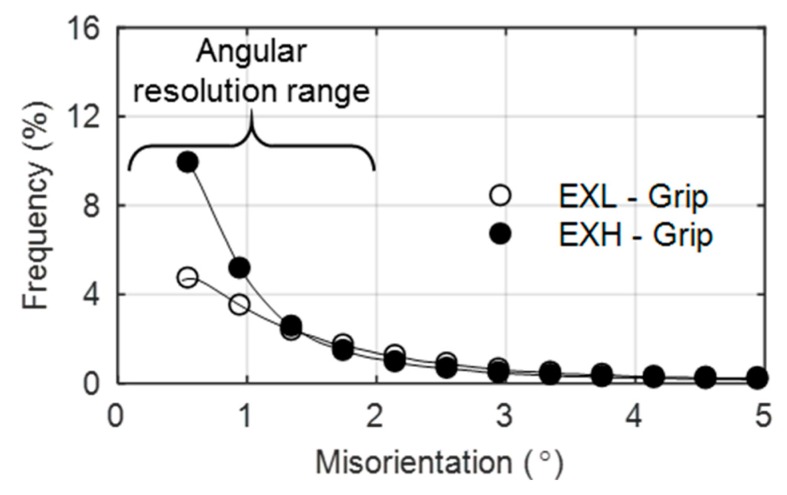
Distribution of misorientation angles (<5°) in EXL and EXH showing a higher frequency of subgrain (<1°) boundaries in EXH grip material.

The misorientation of grain boundaries can also be used as an indicator of creep damage between the material states [18]. Figure 9 shows a comparison of the low angle (<5°) misorientation distributions between EXL and EXH grip regions. 

**Figure 10:**
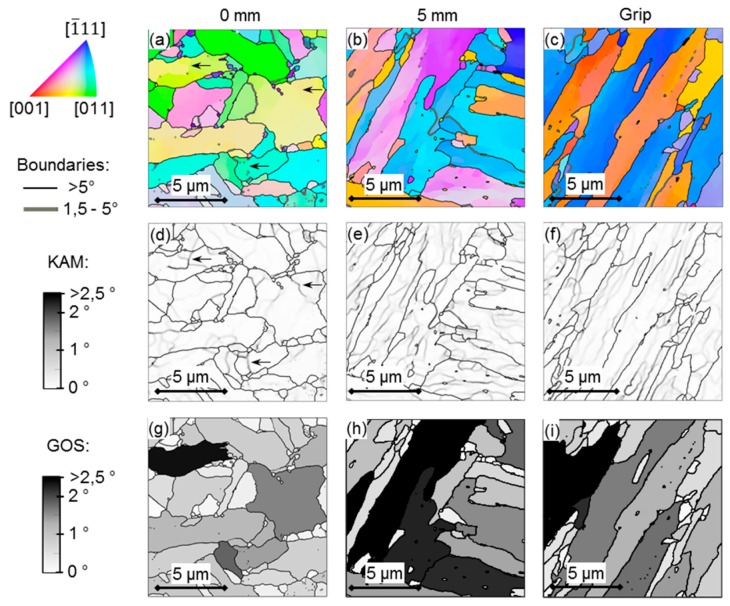
(**a**–**c**) IPF maps showing crystal orientations relative to the sample normal according to the colour key (top left), (**d**–**f**) Kernel Average Misorientation (KAM) maps and (**g**–**i**) grain orientation spread (GOS) maps of areas at 0 mm and 5 mm from the maximum temperature gauge region and at the grip of the EXL material.

**Figure 11:**
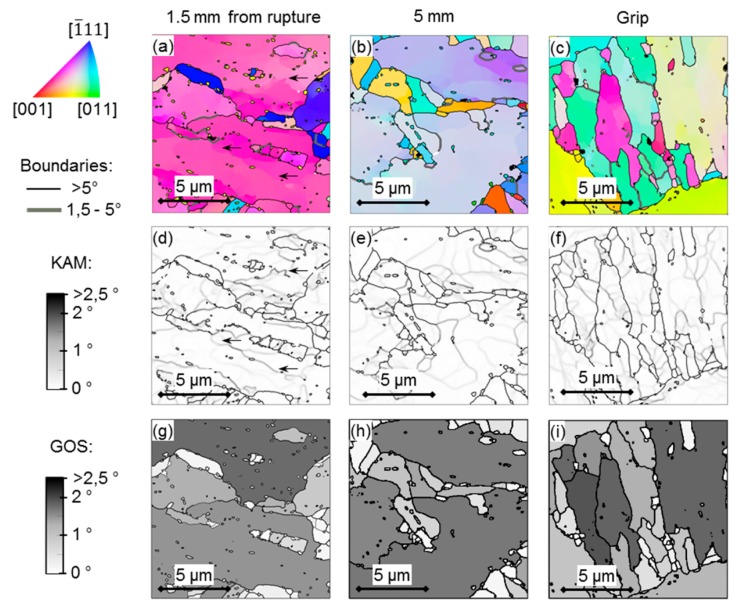
(**a**–**c**) IPF maps showing crystal orientations relative to the sample normal according to the colour key (top left), (**d**–**f**) KAM maps, and (**g**–**i**) GOS maps of areas at 0 mm and 5 mm from the maximum temperature gauge region and at the grip of the EXH material.

An increase in subgrain (<1°) boundaries is noted for the EXH grip material over the EXL material. Increased creep exposure has been found by Aghajani et al. [18] to cause the same result, however it must be emphasised that any measurement of subgrain boundary misorientaiton (<1°) is subject to scrutiny, given that the angular resolution of traditional EBSD setups range from 0.5°–2°, as indicated in Figure 9 [41]. Nonetheless, the increased frequency in subgrain boundaries supports the higher micro-grain boundary density (Table 4) that is noted in the EXH material. No significant differences in the misorientation angles are noted above about 1.5° for the EXL and EXH material. Further investigation was conducted while using the high resolution TKD orientation maps shown in Figure 10 and Figure 11 taken from near the center of the gauge region and location of the maximum temperature of 600 °C (labelled ‘0 mm’ for EXL and ‘1.5 mm from rupture’ for the ruptured EXH specimen), 5 mm offset from the highest temperature location (at approximately 550 °C), and from the grips of the EXL and EXH tested samples, respectively. 

TKD maps were used to investigate another means of assessing creep damage, as proposed by Fujiyama et al. [42], for high Cr steels involving the analysis of Kernel Average Misorientation (KAM), which is the accumulated misorientation angle measured over a kernel of pixels surrounding each measurement point. 

The value is related to intragranular misorientation gradient and it consequently establishes regions of orientation differences that are associated with a dense collection of dislocations present in subgrain boundaries and is sensitive to EBSD step size. Grain orientation spread (GOS) also has been employed [43] as a means of damage assessment that is independent of the step size and it is the average misorientation of each point in a grain from the grain average orientation. This indicator has been reported to be more sensitive to strain accumulation within grain interior than KAM methods yet is susceptible to differences in grain sizes. KAM maps using a kernel size of 9 × 9 pixels^2^ (0.24 × 0.24 µm^2^) are shown in Figure 10d,e,f and Figure 11d–f, together with maps of GOS in Figure 10g–i and Figure 11g–i calculated with a 5° boundary threshold at the three locations, respectively. The arrows in the KAM maps (Figure 10d and Figure 11d) point out examples of boundaries or locations of high densities of dislocations that are not identified as subgrain boundaries (<1.5°) in the corresponding orientation maps (Figure 10a and Figure 11a).

**Figure 12:**
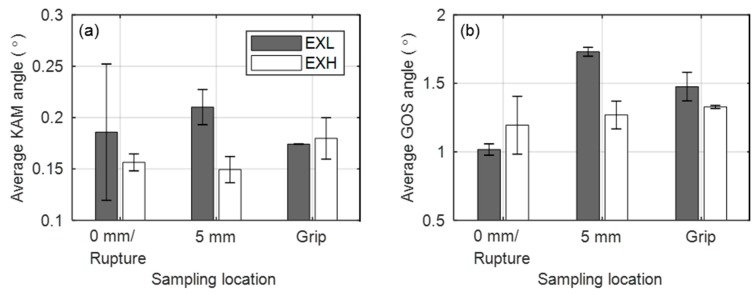
Average (**a**) KAM and (**b**) GOS angles for EXL and EXH material at various sampling locations in the specimen gauge regions.

The average KAM and area-weighted average GOS values calculated from each location of EXL and EXH specimens are shown in Figure 12a,b, respectively. As Fujiyama et al. [42] found that the mean KAM values decrease with increased creep exposure and correlate with a decrease in dislocation density and subsequently hardness, it can be concluded from Figure 12 that EXH demonstrates higher damage accumulation possibly due to competing processes of migration of micro-grain boundaries during grain growth and an increase in frequency of subgrain boundaries, which is also manifested as a reduction in hardness, as shown in Figure 6. 

Interestingly, the highest GOS and KAM values are noted at the 5 mm location for the EXL material. Seeing as the 5 mm sampling location experiences primary creep (see Figure 4c), we expect a large reduction in dislocation densities, as any remaining free dislocations migrate to subgrain boundaries in this regime [33].

However, subgrains are pinned by carbides (that are later shown to be more closely spaced in the EXL material). This causes the internal stain of the grains to increase, which is represented by the higher KAM and GOS values in the EXL material at this location. A strong inflection point is noted in the creep curve of EXL at the 0 mm location (see 223 MPa curve in Figure 5e) similar to that reported for a 15Mo3 material in [44]. This corresponds with a low GOS value at 0 mm, which corresponds to the relief of the internal strain. The EXH material seems to overall have lower KAM and GOS value, which suggests that little resistance is offered in terms of the pinning of dislocations by carbide distribution that would normally increase the internal strain. 

#### 3.3.3. Grain Size Analysis

**Figure 13:**
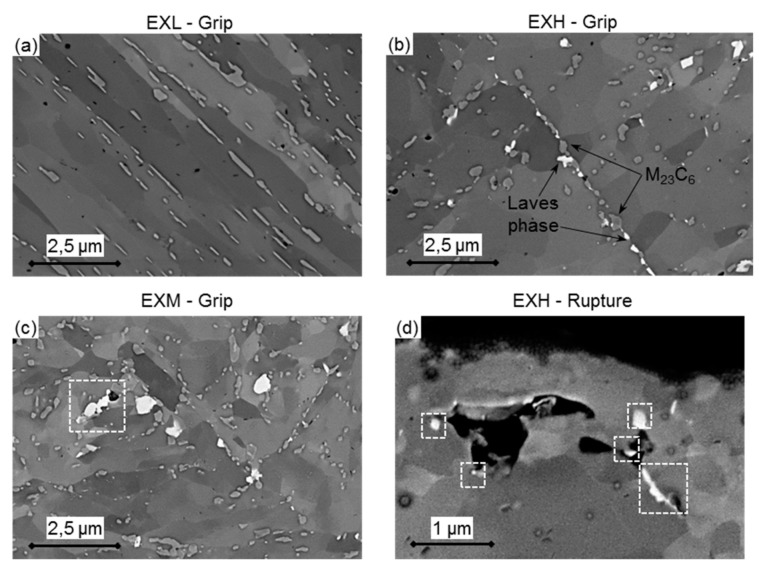
Concentric Backscatter-SEM (CBS-SEM) images of (**a**) EXL, (**b**) EXH and (**c**) EXM grip regions. M_23_C_6_ and Laves phase particles can be preliminarily identified in the images based on the local brightness of features. Laves phase particles are found adjacent to large voids near the (**d**) rupture surface of EXH as outlined in white squares.

Qualitative comparison of micro-grain sizes can be seen in the BS-SEM images in Figure 7a–c, which suggests that EXH has larger, polygonal-shaped grains than the EXL and EXM material. This is as a result of the well-document effect of micro-grain coarsening that occurred during creep exposure in service and serves as microstructural indicator of creep damage, as larger grains offer less pinning resistance to mobile dislocations [45]. Figure 13 shows BS images while using a CBS detector at a 10,000× magnification revealing micro-grains speckled with precipitates (see Section 3.3.4. Precipitate Analysis).

Table 5 summarises the measured micro-grain short widths measured from BS micrographs and reconstructed from EBSD data with a threshold of 5° [46]. Micro-grains are, on average, larger in the EXH material than the materials with lower service exposure when considering the BS micrographs, although the differences are more subtle from EBSD analysis. This suggests that micro-grain growth is only detected at less than 5° misorientations, as the growth of subgrains with low-angle boundaries is one of the main contributors to creep damage. However, this is difficult to detect due to subgrain boundary misorientations (~1°) that are within the angular resolution of the EBSD technique. Relatively large micro-grain sizes in the EXL material also support evidence of a decrease in boundary density (Table 4). The tendency to more equiaxed grains is evident from the larger micro-grain shape factors (ratio of minor to major ellipse axis) in EXH and EXM in comparison to EXL which is also substantiated by the observation of polygonised grains (Figure 13b,c).

#### 3.3.4. Precipitate Analysis

Typically, two main types of precipitates form in X20 following heat treatment: M_23_C_6_ carbides (where M = Cr, Fe, Mo) and VX carbonitrides (where X = C, N). Service creep exposure promotes the morphology evolution of these particles through diffusion processes and also results in the formation of new Laves phases (Fe,Cr)_2_(Mo), which leads to a reduction in solid solution and precipitation strengthening of the material [18]. Besides differing in chemical composition, M_23_C_6_ and Laves phase particles have been reported [47,48] to be larger (with diameters ranging from 200–400 nm) than the VX counterparts and can be identified through resolutions that are typical to CBS-SEM imaging along with the micro-grain detail, as shown in Figure 13. VX particles, however are too small (<100 nm) to be identified from CBS-SEM images and are instead captured by EFTEM analysis.

**Figure 14:**
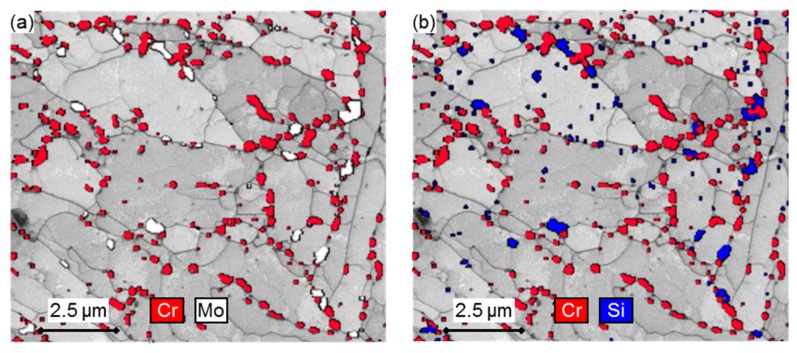
EDS maps of (**a**) Cr and Mo and (**b**) Cr and Si overlaid over Transmission Kikuchi Diffraction (TKD) band contrast maps in EXH material, indicating the location of carbides (Cr) and Laves phase particles (Mo and Si) along micro-grain boundaries.

Several precipitates are seen decorated along micro-grain boundaries in Figure 13, with varying degrees of brightness based on their constituent atomic numbers. Seeing as the atomic numbers of Mo and Cr is 42 and 24, respectively, it is expected to identify Laves phase and Cr-rich M_23_C_6_ particles from BS map features with corresponding brightness levels with the former being the brightest (Figure 13b for example). An overlay of EDS maps over TKD band contrast maps in Figure 14a indicates that the Laves phase are concentrated regions of Mo, which also correspond to regions of Si, as shown in Figure 14b. This is in agreement with Isik et al. [49] and Aghajani et al. [18], who found that Si promotes Laves phase nucleation. 

**Figure 15:**
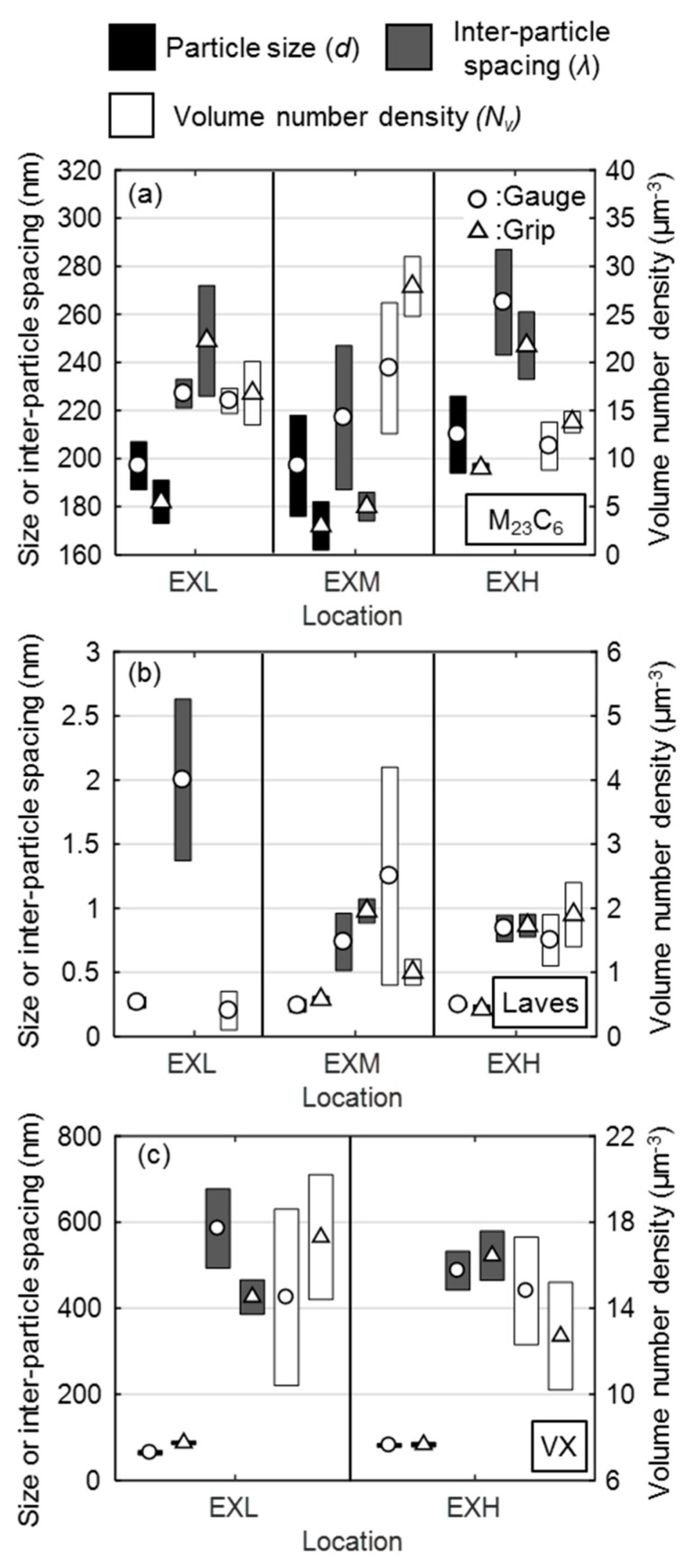
Error bar plots of particle diameter, inter-particle spacing and volume number density of (**a**) M_23_C_6_ (EXL-2570, EXM-2467, EXH-2560 particles), (**b**) Laves phase (EXL-40, EXM-226, EXH-364 particles), and (**c**) VX precipitates (EXL-348, EXH-345 particles) in the gauge (circles) and grip (triangle) regions of the EXL and EXH material.

The EXL material demonstrates stringers of M_23_C_6_ particles (notably Figure 13a) along micro-grain boundaries that appear to be more broken up in the EXM and EXH material (Figure 13b,c). Evidence of cavity growth near larger Laves phase particles was found in EXM (as seen in Figure 13c, outlined by a white square) and EXH. Specifically, large voids surrounded by Laves phase clusters were noticed near the rupture surface of an EXH specimen in Figure 13d. Figure 14 also indicates that Laves phase predominantly nucleates adjacent to Cr-rich carbides and along micro-grain boundaries. The mean particle diameter, volume number density, and inter-particle spacing [32] of M_23_C_6_ and Laves phase particles are compared in the error bar plots in Figure 15a,b, respectively.

Although no differences are expected between the grip and gauge regions on account of the short nature of the accelerated creep tests, the heterogeneous distribution of the low fraction precipitates (in particular Laves phase) require analysis of both regions to more holistically quantify the precipitate details.

**Figure 16:**
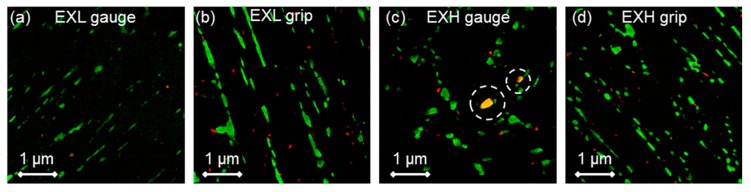
Composite Cr (green) and V (red) elemental maps obtained from Energy-Filtered Transmission SEM (EFTEM) of (**a**,**b**) EXL and (**c**,**d**) EXH gauge and grip regions, respectively. Z phase particles are indicated by the circle outlines in (**c**).

**Figure 17:**
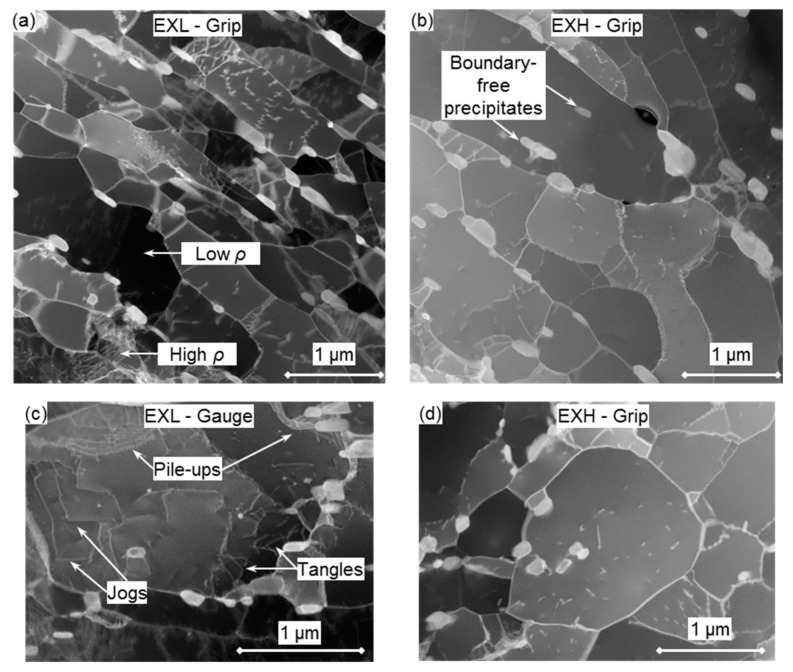
Annular Dark Field Scanning TEM (ADF-STEM) micrographs of (**a**) EXL, (**b**) EXH grip regions, (**c**) various dislocation interactions at the gauge center of EXL material, and (**d**) an equiaxed micro-grain in the EXH grip region. Regions of high and low concentrations of dislocations *ρ* are indicated in (**a**).

From these measurements, three main conclusions can be drawn. Firstly, EXH, on average, demonstrates larger M_23_C_6_ particles (as illustrated in Figure 16), which are spaced further apart with a lower number density. This manifests in a weakening of the pinning effect of micro-grains by precipitates (as later deduced from the ADF-STEM micrograph in Figure 17b, which shows the increased presence of interior precipitates and the loss of the close distribution of particles along boundaries that are present in EXL shown in Figure 17a). Secondly, little difference is found between particle parameters in the grip and gauge region given the short duration of the accelerated creep tests and the slow rate of diffusion-related precipitate growth under creep conditions [18,23]. In fact, any differences can be attributed to the heterogeneous nature of microstructural features throughout the pipe material. However, it must be noted that no Laves phase was found in the sampled grip region of the EXL material, suggesting a less frequent presence of this particle in EXL. Indeed, similar Laves phase inter-particle spacings were measured in the EXM and EXH materials. Lastly, a higher number density and smaller inter-particle spacing of M_23_C_6_ particles is found in the EXM material. A source of error comes from falsely identifying Z phase (CrVN) as M_23_C_6_ particles, although the effect on these measurements is considered to be negligible on account of the low Z phase fractions that are present in X20 [18]. 

Composite Cr (green) and V (red) EFTEM maps are shown in Figure 16 for the EXL (a,b) and EXH (c,d) gauge and grip regions, respectively. The VX particles appear to be dispersed in the matrix with no preferential alignment, as evident for the larger Cr-rich particles. The extraction of VX particle parameters from thin foil samples while using this technique is challenging due to the degradation of the signal-to-noise ratio from the iron matrix (most notably in Figure 16a) as well as the low sample density. However, small differences between the EXL and EXH materials are still evident in the calculated parameters that are shown in Figure 15c. Most notably, the coarsening of VX particles with a concomitant increase in inter-particle spacing and decrease in number density is found in the EXH material as compared to the EXL counterpart. However, differences in the gauge region are not as obvious. It has been suggested that, since no significant changes in VX precipitate morphology is found during long-term creep testing of X20, these particles play a smaller role than M_23_C_6_ precipitates in micro-grain stability [18,50].

Z phase particles are highlighted in the EXH gauge region (Figure 16c) identified as overlapping regions of the Cr and V maps. Observation of this phase suggests that EXH shows increased dissolution of the creep strengthening VX particles due to the growth of large Z phase particles, resulting in decreased creep strength and increased creep damage [11]. 

#### 3.3.5. Dislocation Density and Substructure

Qualitative differences in dislocation densities and substructure are shown in ADF-STEM micrographs in Figure 17a,b for EXL and EXH grip regions, respectively. A heterogeneity in micro-grain-interior dislocation densities is noted in the EXL material with some micro-grains displaying a higher concentration of dislocations than others. Lower densities are noted in the larger micro-grains in the EXH material. The presence of larger and more polygonised grains that are shown in Figure 17d in the EXH state reinforces previous observations (Table 5).

Comparisons between the respective grip and gauge regions of the EXL and EXH material states reveal substructure and damage features that are representative of different mechanisms. Prior to testing (best represented by the grip regions), the microstructural features reflect the pre-existing effects of the diffusional creep regime that is typically encountered in service conditions in power plants [28]. Following accelerated creep testing (represented by the gauge microstructures), tangles, pile-ups, and jogs of dislocations are evident in both material states, yet are more common in the EXL material, as indicated in Figure 17c, due to higher dislocation densities within the micro-grains. Pile-ups are evident at some micro-grain boundaries and at large precipitates. These interactions are not noted as extensively in the grip regions, which suggests that testing is within the dislocation climb deformation regime, as suggested by changes in the gauge microstructures [51]. 

## 4. Discussion

### 4.1. Influence of Cavity Damage

The impact of the difference in cavity damage can be observed by the shorter rupture times, high strain rates, and tertiary-dominated character of the EXH accelerated creep curves (Figure 5c), lending credence to the use of cavity density as a damage class indicator [21]. The effect of the high number of cavities on the creep rupture mode can be observed through the calculation of creep damage tolerance factor λ = $\varepsilon_{r}/\left({\dot{\varepsilon }}_{m}t_{r}\right),$ where $\varepsilon_{r}$ is the strain at rupture, $t_{r}$ is the time to rupture, and ${\dot{\varepsilon }}_{m}$ is the minimum creep rate. Values around λ = 1 indicate brittle fracture modes, whereas values in excess of 2.5 indicate a combination of plastic deformation from necking, transgranular cracking, and microstructural degradation (for λ > 5) [52]. For the current accelerated creep tests, the factor was found to be around 3–3.5 for EXH and ~4 for the virgin material. Similar trends in degradation of creep ductility were observed by Roy et al. [53] for the ex-service P-22 alloy. The lower λ value suggests that damage due to necking and transgranular crack growth from plastic deformation is more likely to occur in EXH than the virgin material in the current accelerated tests due to the presence of closely spaced cavities. Evidence of this is shown in Figure 7e, where large connected cavities with traces of aluminum inclusions were found to coalesce perpendicular to the loading direction near the ruptured surface of an EXH specimen at 600 °C and 223 MPa.

Moreover, cavities that are associated with large Laves phase particles were found in the EXM and EXH material, which suggests a correlation of higher cavity densities with higher Laves phase number densities in a similar manner to that Zhang et al. presented [54] on F91 steel. As EXL is expected (from replica analysis) to outlive EXM material, the current findings suggest that other microstructural features besides cavity density are responsible for the accelerated creep rates of EXL. 

### 4.2. Calculation of Deformation Mechanism Parameters

As temperature and stress is varied in traditional creep experiments, deformation mechanisms at which creep deformation takes place also alters. For instance, at low stress levels at high temperatures, the rate-controlling mechanism is usually diffusional creep [12]. In accelerated creep testing (conducted at intermediate stress and temperature levels), dislocation creep is assumed to dominate based on observations in similar particle-strengthened steels. A typical description of the dislocation creep rate behaviour is given by the Bird-Mukherjee-Dorn (BMD) expression, modified for threshold stress [12]:

(1) $${\dot{\varepsilon }}_{m}=A\frac{D_{o}Eb}{kT}{\left(\frac{\sigma_{x}-\sigma_{o}}{E}\right)}^{n}\exp\left(-\frac{Q}{RT}\right)$$

where ${\dot{\varepsilon }}_{m}$ is the steady state creep rate (minimum creep rate in the case of 9%–12% Cr steels), A is a mechanism-dependent constant, E is the temperature-dependent Young’s modulus, k is the Boltzmann constant, b is the Burger vector, R is the universal gas constant, $\sigma_{x}$ is the average longitudinal stress, as established by FEM, T is the applied temperature in Kelvin, $D_{o}$ is the diffusivity frequency factor, Q is the mechanism-dependent activation energy, n is the stress exponent, and $\sigma_{o}$ is the threshold stress. 

Different deformation mechanisms are elucidated by the values of the parameters Q and n. Additionally, $\sigma_{o}$ can also be investigated as a point of comparison between the two aged materials. $\sigma_{o}$ is theorised to be necessary to account for the stress that is required to overcome departure side pinning of dislocations on incoherent particles [11]. Without the stress adjustment, high stress exponents and physically impossible activation energies above the value for bulk diffusion in α-Fe are calculated [12]. 

By following the procedure that was laid out by Shrestha et al. [12], to determine Q, n, and $\sigma_{o}$ while using the modified BMD relation, mechanism differences (if any) can be identified between EXH and EXL material. Various models [12] were developed to predict the stress value to show a dependency on precipitate size and inter-particle spacing. As the microstructural investigation in Section 3.3.4. Precipitate Analysis has revealed that EXH and EXL possess different precipitate morphologies that will theoretically manifest in different threshold stress values. 

**Figure 18:**
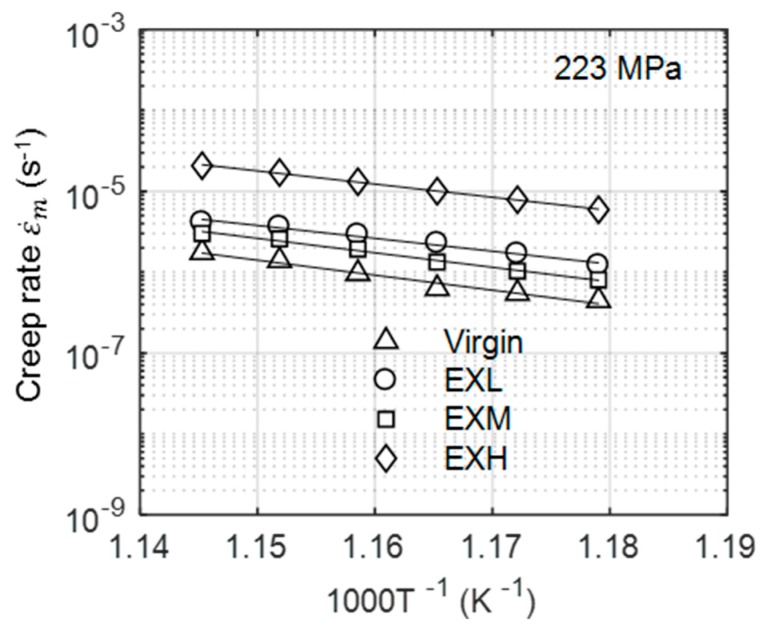
Logarithmic creep rate versus inverse temperature plots for all material states at 223 MPa.

The determination of activation energy requires the variation of minimum creep rate with temperature. The DIC technique that was used in this work allows for the measurement of accelerated creep curves at multiple temperatures from a single sample within a uniform stress field. From each curve, the minimum creep rate was extracted and plotted on a semi-logarithmic plot in Figure 18 for each material state at 233 MPa. As deduced from the gradients of each plot, Q appears to approximately equal between all of the materials. Prior to threshold stress correction, an activation energy of 335 ± 33 kJ/mol for EXL and 311 ± 43 kJ/mol for EXH is revealed across the different stress states. These values exceed the activation energy for bulk diffusion in α-Fe (240 kJ/mol) [12]. Similarly, the n values were calculated to be 10.2 ± 0.7 for EXL and 11.4 ± 0.3 for EXH across the investigated temperature range. The nearly parallel curves in Figure 18 signify that no change in deformation mechanism occurs over the range of testing conditions and materials. However, such relatively high values for the BMD parameters indicate that compensation for threshold stress is necessary. 

Threshold stress correction was performed while using the procedure that was outlined in [12], which involves plotting the creep rates to the power of an assumed inverse stress exponent against modulus-normalised applied stress. The exponent *n* was varied between 3 (viscous glide), 5 (high temperature dislocation climb via lattice diffusion), and 7 (low temperature dislocation climb via pipe diffusion) until the best fit is obtained for all temperatures, and the temperature-dependent $\sigma_{o}$ is then obtained from the intercept of the best-fit curve with the stress axis at a zero creep rate value. Data from square Gleeble specimens are included. For both EXL and EXH materials, stress exponents of 7 achieved a correlation coefficient of ≥0.97, which suggested that dislocation climb via pipe diffusion is the dominant deformation mechanism. Similar results were found for virgin and retired X20 investigated by El Rayes and El-Danaf [10]. Based on the resulting exponents, $\sigma_{o}$ values of approximately 75–85 MPa and 85–90 MPa were calculated for EXL and EXH, respectively, at temperatures that ranged from 600–575 °C. This suggests that EXL demonstrates similar resistance to dislocation climb from precipitates as the EXH counterpart within experimental scatter. In order to relate the experimental $\sigma_{o}$ with the measured particle distributions, a model prediction that was developed by Arzt et al. [55] is employed, given in Equation (2), while using a Taylor factor of 2.73 to convert from shear to normal stresses:

(2) $$\sigma_{o}=2.73G\frac{b}{\lambda }\sqrt{1-k_{A}^{2}}$$

This model considers the detachment stress that was involved in freeing dislocations from the particle-dislocation attraction to be synonymous with the threshold stress and it was recently shown by Zhao et al. [11] to work well in predicting creep rates at intermediate stresses for Grade 91 ferritic steels. The model variables and constants are given in Table 6, including the inter-particle spacing that is associated with the VX particles measured in this study. As M_23_C_6_ are considered to stabilise micro-grain boundaries, the VX particles that are dispersed within the grain matrix are the main obstacles to dislocation motion and are therefore employed in this model. 

Using this model, values of 97 (71) MPa and 79 (85) MPa were predicted while using inter-particle spacing values that were measured for VX precipitates from EXL and EXH grips (gauge values are supplied in brackets), respectively.

**Figure 19:**
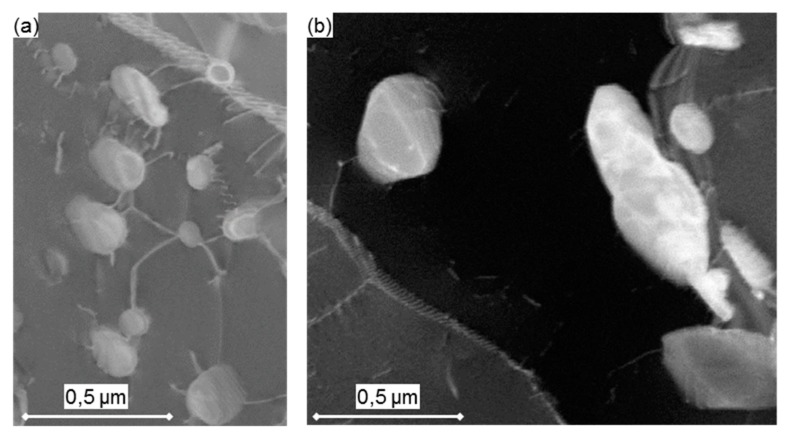
ADF-STEM images showing (**a**) multi-particle pinning of dislocations by M_23_C_6_ particles closely spaced together in the EXL material and (**b**) dislocations bypassing larger precipitates spaced further apart in the EXH material.

It is evident that the increased precipitate diameters and, consequently, longer inter-particle spacing in EXH has resulted in a lower threshold stress than for the EXL material, as predicted from Equation (2) while using the grip values. Lower values that were predicted from gauge measurements in the EXL material are due to higher amount of scatter present in these EFTEM-based measurements (seen in Figure 15c). Although EXH demonstrated close agreement with the calculated values, the model over-estimated the threshold stress for EXL by <20% as compared to the experimental results. This could be due to several reasons, including measurement scatter, due to the lack of particle statistics that are associated with small EFTEM sampling windows and difficulty in determining the model constants [57]. There is also evidence that the VX particles play a smaller role as compared to M_23_C_6_ on creep strength enhancement [18]. However, the same model cannot be applied to M_23_C_6_ precipitates as dislocation climb kinetics are altered with smaller inter-particle spacing to radius ratios [55]. Both the model and analytical method indicate a similar weakening of the particle-strengthening effect in EXL as noted in EXH, although a clear comparison is restricted by experimental scatter in the creep strain and microstructural data. Evidence of the prevailing dislocation-particle interaction is shown in Figure 19a for the EXL material, where dislocations are trapped by closely spaced particles. On the other hand, a larger spacing between precipitates as well as the increased presence of large (>0.25 µm diameter) Laves phase particles in EXH material offers less resistance to dislocation migration, as shown in Figure 19b.

**Figure 20:**
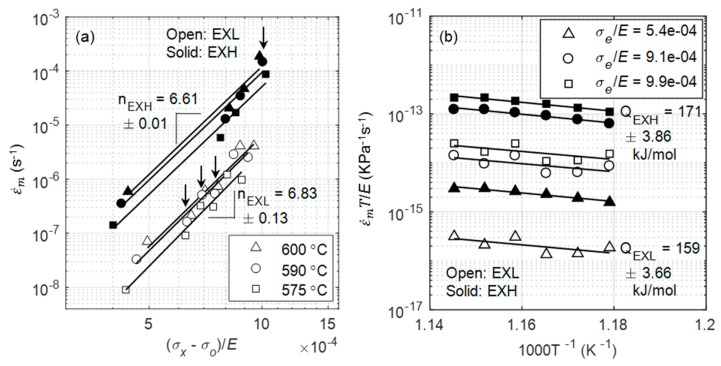
(**a**) Calculation of stress exponents from log-log plot of minimum creep rate versus normalised effective stress with data from square specimens indicated by arrows; (**b**) Calculation of activation energies for EXL (open symbols) and EXH (solid symbols) at three modulus compensated stresses.

Following threshold stress correction, the activation energies and stress exponents were calculated, as shown in Figure 20. Data from lower temperatures (<575 °C) were excluded for the following analysis, as creep curves failed to reach the secondary creep stage within the testing time, which is evident by a lack of minimum points in Figure 4f. Further verification of the stress exponent of 7 is obtained by using the threshold stress that was previously calculated in a double logarithmic plot of minimum creep rate versus normalised effective stress in Figure 20a, where the gradient is calculated as 6.61 ± 0.01 and 6.83 ± 0.13 across all temperature values for EXL and EXH, respectively. 

Activation energies were calculated from three modulus normalized effective stress ($\frac{\sigma_{e}}{E}=\frac{\sigma_{t}-\sigma_{o}}{E}$) of 5.4 × 10^−4^, 9.1 × 10^−4^, and 9.9 × 10^−4^, and they were found to be 159 ± 4 kJ/mol and 171 ± 4 kJ/mol for EXL and EXH, respectively (Figure 20b). Calculated energy values for EXL and EXH are close to 60% of the theoretical value of lattice self-diffusion in α-Fe (0.6 × 250 kJ/mol = 150 kJ/mol), which is expected for low temperature climb as a governing mechanism, in which the pipe diffusion of atoms occurs through dislocation cores [10].

**Figure 21:**
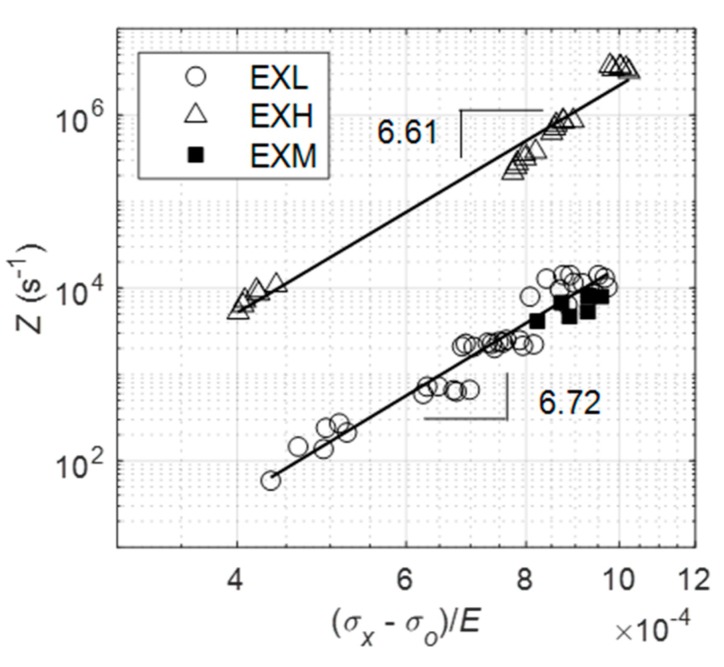
Plot of Zener–Hollomon parameters of EXL and EXH materials. A single stress state for EXM is included for comparison with EXL.

As a final comparison between the two aged materials, the data for each condition are collected onto a single reference plot, as shown in Figure 21 while using the Zener–Hollomon parameter [10]:

(3) $$Z=\;{\dot{\varepsilon }}_{m}\exp\left(\frac{Q}{RT}\right)=A{\left(\frac{\sigma_{x}-\sigma_{o}}{E}\right)}^{n}$$

Data from both EXL and EXH each fall onto a straight line on the double logarithmic plot, the gradient of which closely corresponds with the stress exponents that were previously calculated in Figure 20a and they are represented by equations of 2.56E24${\left(\frac{\sigma_{x}-\sigma_{o}}{E}\right)}^{6.72}$ and 1.5326${\left(\frac{\sigma_{x}-\sigma_{o}}{E}\right)}^{6.61}$ for EXL and EXH, respectively. These results are similar to findings by El Rayes and El-Danaf [10] for as-produced and ex-service 12% Cr steel while using hot tensile tests.

Z values from a single stress state for the EXM material lies close to the EXL data, stemming from similar creep rates between the two material states. This implies that the Z parameter that was obtained while using the proposed accelerated creep technique can be used to establish the relative damage between service-exposed X20 as an alternative to other techniques, such as hardness testing, which would otherwise not reveal a difference in damage. 

### 4.3. Comparison of Mechanical and Microstructural Damage Indicators

This technique has proven that differences in levels of damage of service-exposed material can be identified while using both mechanical testing methods that are based on DIC and microstructural analysis. The mechanical testing-derived parameters, specifically the Zener–Hollomon parameter, are shown to be a simpler indictor of damage in comparison to microstructural factors, which are often more complicated to analyse. This is due to the complex interactions between precipitates and micro-grain coarsening (Figure 13); dislocation pinning and precipitates (Figure 19); dislocations and micro-grain boundaries (Figure 17c); and, between different types of precipitates (Figure 14 and Figure 16). Slow evolution of some microstructural features, depending on the point of life exhaustion, result in subtle differences between the ex-service materials (as shown for characteristic boundary features and EBSD-based micro-grain size in Table 4 and Table 5, respectively) and further exacerbates the difficulty of using microstructural analysis as a form of damage measurement [21]. There is a high scatter associated with the microstructural statistics possibly due to different starting microstructures, alloy compositions for the EXL and EXH material, and spatial heterogeneity of some microstructural features. For instance, the difference in misorientaion indicators, such as KAM and GOS, is not as obvious if untested material at the grips were analysed—indicating that the testing manifests differences between EXL and EXH in the gauge regions. Finally, it should be noted that small window analysis is very sensitive to non-uniform spread of microstructural features in the sampled field, resulting in large standard deviation values, as shown in Figure 12.

In this case, the close similarity in damage between EXL and EXH was initially revealed through hardness measurements (Figure 6). Interestingly, the EXL hardness measurements are lower than those for the EXM specimen and closer to that of EXH, both of which have higher cavity densities. Thus, the lower hardness can be attributed to features of microstructural degradation (such as larger micro-grains and/or precipitates) similar to that displayed by EXH rather than due to the presence of cavities [6].

**Figure 22:**
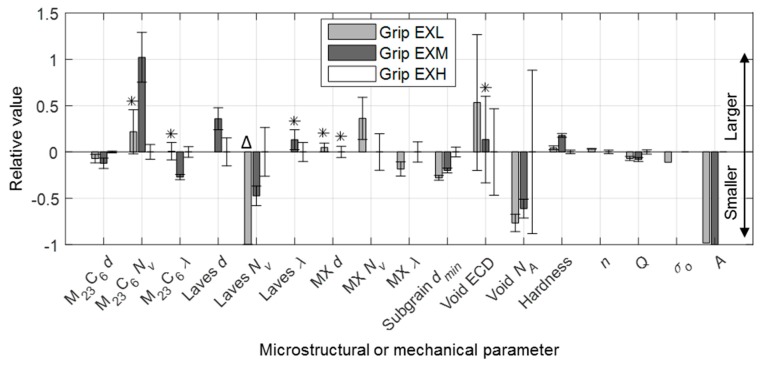
Relative differences in microstructural parameters indicating damage using the EXH grip region as a baseline. Note: *d* = diameter, *d_min_* = minor diameter, *N_v_* = volume density, *λ* = inter-particle spacing, *N_A_* = area density, *n* = stress exponent, *Q* = activation energy, *σ_o_* = threshold stress, *A* = Zener–Hollomon pre-exponential. Asterisks (*) indicate features that are not statistically different from the baseline. Δ indicates that no Laves phase was recorded in the EXL grip region

Based on these grounds, key mean microstructural parameters are compared via unpaired *t*-testing with a 0.05 significance value to indicate statistically significant differences between EXL, EXM, and EXH grip regions in Figure 22 while using values of the latter region as a baseline. Mechanical-based parameters, such as BMD equation parameters, threshold stresses and Zener-Hollomon pre-exponentials A, are also included along with the traditional means of damage assessment using cavity densities. 

The main limitation in the current analysis is that each stress condition requires a separate specimen. This means that any stress-based analysis (such as for determining stress exponents and threshold stresses) is specimen intensive. However, in keeping with the requirement for ex-service material economy in power plant applications, the current work that employs variable temperature profiles is better suited to temperature-based calculations (such as for activation energy and the Zener-Hollomon parameter). Using the current testing parameters, Figure 22 demonstrates that no notable difference is evident between the ex-service materials in terms of stress-based parameters. For this reason, less onus is placed on these parameters as a method of damage characterisation.

The most prominent differences in microstructural damage indicators is noted in micro-grain width (measured from BS-SEM), as well as the precipitate distribution, specifically Laves phase (between EXL and EXM/EXH) and M_23_C_6_ (between EXM and EXL/EXH). The micro-grain width shows increasingly coarser, polygonised grains with damage level, correlating somewhat with cavity density measurements. With no significant amounts of Laves phasesbeing observed in the EXL grip, one contributing factor to increased damage in EXH is the solid solution depletion of Mo and Cr due to the formation of Laves phase ((Fe,Cr)_2_Mo) and the growth of M_23_C_6_ carbides. This effect appears to outweigh any potential benefit gained from particle strengthening [18]. The relative ability of different precipitates to pin micro-grain boundaries is established by considering their respective normalised Zener pinning pressure, given as $P_{z}/\gamma =3f/d$ [58], where $P_{z}$ is the Zener pinning pressure, γ is the boundary surface energy per area, f is the particle volume fraction and d is the particle diameter. In the EXH gauge region, M_23_C_6_ and Laves phase precipitates result in an average normalised pressure of 0.74 and 0.13 µm^−1^, respectively. The corresponding values in the EXL gauge region are 0.97 and 0.045 µm^−1^, respectively. Therefore, Laves phase is shown to be ineffective in pinning micro-grains, providing a 5–20 smaller pinning pressure as compared to M_23_C_6_ particles. Additionally, as Laves precipitation is known [49] to often occur adjacent to M_23_C_6_ particles (also found in this work in Figure 13b and Figure 14), the Zener pinning pressure that is responsible for micro-grain boundary pinning is reduced as the effective precipitate size increases. Such clustering has also been found in previous research to cause a reduction in yield strength of the material, especially when the particle sizes exceed 0.2 µm (similar to sizes obtained in this study) [59]. Although it has been shown that solid solution depletion of Mo takes a minor role in overall creep damage in some 9%–12% Cr steels [18], it is considered here together with larger micro-grain sizes to be the principle difference between EXL and EXH besides cavity density. Traces of Z phase that are found in the EXH material could also suggest higher damage levels than in EXL, although no conclusions can be drawn from the small amount observed. 

The deformation resistance of EXM revealed from the deformation tests, however, cannot be explained in the same manner as it has similar Laves phase inter-particle spacing to that of EXH. Instead, a dense M_23_C_6_ particle distribution is the only feature investigated that explains the enhanced strength of EXM, with $P_{z}/\gamma$ values of 1.29 µm^−1^ in the grip region. Previous investigations [18,50] emphasised the hardening effect of these carbides from stabilising the substructure and pinning boundaries. These microstructural results, together with the deformation measurements, therefore suggest that EXM has a similar amount of remnant creep strength as EXL, despite the higher category of damage based on cavity density. This highlights the benefit of exploring alternative damage indicators, such as the Zener–Hollomon parameter and precipitate densities, in the assessment of service-exposed X20. Creep strain and rate data that were used in this work as well as microstructural statistics are provided in an online repository [60]. 

## 5. Conclusions

The presented work allows for the comparative damage assessment of ex-service (EXL, EXM, and EXH—low, medium, and high damaged) X20 piping material by comparing the deformation behaviour of the material states through DIC-based mechanical testing and applying advanced electron microscopy to elucidate the link with microstructural features. Both techniques correlate well when considering the Zener–Hollomon parameter and Laves phase and carbide distributions. In summary, the following conclusions are highlighted:

1. Multiple accelerated creep curves at several temperatures ranging from 550–600 °C are extracted from single samples of virgin and ex-service 12% Cr steam piping steel using the full-field capabilities of DIC. Increasingly higher creep strains and rates were measured for virgin, EXM, EXL, and EXH material, respectively.
2. Orientation mapping revealed higher boundary densities, higher frequencies of small angle (<1°) boundary misorientation angles and smaller KAM and GOS values for the EXH material. Micro-grain sizes determined from EBSD were found to be very similar between the two materials, whereas BS-SEM identified larger, near-equiaxed grains in the higher damage materials.
3. An increase in the mean M_23_C_6_ size and Laves phase size and spacing was noted in the EXH material and no Laves phase was found in the sampled EXL grip region. The differences in the VX precipitate distributions were subtle.
4. Following threshold stress correction using the BMD formulation, stress exponents of 7 and activation energies of 159 ± 3.66 and 171 ± 3.86 kJ/mol similar to that of pipe diffusion were calculated for the EXL and EXH material, respectively, pointing to low temperature dislocation climb as a potential deformation mechanism.
5. Lower threshold stresses computed while using microstructural means suggest that EXH material has a reduced ability of pinning dislocations as also identified qualitatively by dislocation-denuded micro-grains and coarse precipitates in ADF-STEM analysis. Analytical calculations suggest similar threshold stresses between the EXL and EXH material within the experimental scatter.
6. Higher Zener–Hollomon values were found for EXH compared to the EXL and EXM, serving as a reliable damage indicator based on DIC measurements.
7. Extensive reduction in solid solution strengthening and pining of precipitates through Laves phase and M_23_C_6_ coarsening is suggested as the main form of microstructural degradation of EXH over the other material states.
8. Slower creep rates, relatively low Zener–Hollomon values, and dense M_23_C_6_ distributions for the EXM material suggest a remnant creep life close to that of EXL, despite the higher cavity density count and resulting damage category.

Future work utilising the full-field capabilities of DIC opens the possibility of measuring multiple creep curves at multiple stresses using a specialised geometry during traditional creep testing at constant temperature [15]. This will allow comparative damage identification from ex-service material across different deformation mechanism regimes through the more convenient calculation of stress exponents and threshold stresses from a single sample.

## Figures and Tables

**Table 1:** Alloying elements of X20 (X20CrMoV12-1) used in this study in wt % (balance iron).

C	Si	Mn	P	S	Cr	Mo	Ni	V
0.21	0.19	0.58	0.015	0.004	11.6	0.88	0.76	0.25

**Table 2:** Details of investigated service-exposed X20.

Ex-Service Material Designation	Reported Operating Temperature (°C)	Reported Operating Pressure (MPa)	Service Time (h)	Cavity Density from Replicas (mm^−2^)
EXL (Low ex-service damage)	545	17.0	130,000	60–90
EXM (Medium ex-service damage)	545	19.4	130,000	~200
EXH (High ex-service damage)	543	18.1	156,000	220–690

**Table 3:** Range of actual stress and triaxiality ratios over ROI (Region of Interest) at three applied stresses.

Applied Stress (MPa)	Actual Stress Range *σ_x_* (MPa)	Triaxiality Ratio Range
200	145–150	0.315–0.350
250	210–212	0.321–0.357
260	224–228	0.321–0.357

**Table 4:** Comparison of boundary length per unit area of prior-austenite grain constituents between EXL and EXH.

Material	Boundary Density (µm^−1^)		
-	Micro-Grain	Block	Packet
EXL	1.39 ± 0.05	1.10 ± 0.01	0.30 ± 0.06
EXH	1.47 ± 0.05	1.13 ± 0.02	0.40 ± 0.01

**Table 5:** Comparison of average micro-grain width measured from CBS-SEM and EBSD maps between EXL, EXM, and EXH materials (95% confidence indexes are provided).

Specimen	EXL Grip (BS-936 Grains EBSD-5144 Grains)	EXM Grip (BS-1013 Grains)	EXH Grip (BS-551 Grains EBSD-5666 Grains)
Micro-grain width from CBS-SEM (µm)	0.54 ± 0.02	0.60 ± 0.02	0.75 ± 0.04
Shape factor from CBS-SEM	0.52 ± 0.18	0.57 ± 0.18	0.57 ± 0.19
Micro-grain width from EBSD with boundaries > 5° (µm)	0.65 ± 0.01	-	0.67 ± 0.01

**Table 6:** Model inputs for the equation of threshold prediction by Arzt et al. [55].

Symbol	Description	Value	Reference
G	Shear modulus at 600 °C	63.5 GPa	[56] (pp. 5.1–5.3)
b	Burger vector	2.5 × 10^−10^	[18]
λ	Inter-particle spacing of VX precipitates	585 ^1^/426 nm—EXL gauge/grip 487/522 nm—EXH gauge/grip	Section 3.3.4. Precipitate Analysis -
*k_A_*	Detachment factor model constant	0.3	[12]

^1^ Large scatter in VX inter-particle spacing measurements from EFTEM micrographs.
